# Advances of Apetala2/Ethylene Response Factors in Regulating Development and Stress Response in Maize

**DOI:** 10.3390/ijms24065416

**Published:** 2023-03-12

**Authors:** Huanhuan Qi, Kun Liang, Yinggen Ke, Jing Wang, Pingfang Yang, Feng Yu, Fazhan Qiu

**Affiliations:** 1 State Key Laboratory of Biocatalysis and Enzyme Engineering, School of Life Science, Hubei University, Wuhan 430062, China; 2 National Key Laboratory of Crop Genetic Improvement, Huazhong Agricultural University, Wuhan 430070, China

**Keywords:** maize, AP2/ERFs, development, stress, homolog genes, interacting proteins

## Abstract

Apetala2/ethylene response factor (AP2/ERF) is one of the largest families of transcription factors, regulating growth, development, and stress response in plants. Several studies have been conducted to clarify their roles in *Arabidopsis* and rice. However, less research has been carried out on maize. In this review, we systematically identified the *AP2/ERFs* in the maize genome and summarized the research progress related to *AP2/ERF* genes. The potential roles were predicted from rice homologs based on phylogenetic and collinear analysis. The putative regulatory interactions mediated by maize AP2/ERFs were discovered according to integrated data sources, implying that they involved complex networks in biological activities. This will facilitate the functional assignment of AP2/ERFs and their applications in breeding strategy.

## 1. Introduction

Plant growth, development, and response to environmental stimulus were regulated through highly dynamic metabolism. These metabolic processes were mediated by regulons such as transcription factors (TFs), which play central roles in regulating biological activity. The Apetala2/ethylene response factors (AP2/ERFs) family of TFs contains the AP2/Ethylene Responsive Element Binding Factors (EREB) domain, comprising 40–70 conserved amino acids that are involved in DNA binding [1,2,3,4]. Functional analysis of AP2/ERFs in *Arabidopsis* and rice has documented that they were key regulators of stress and hormone response, affecting plant survival under normal and stressful conditions [5,6]. Diverse mechanisms including transcriptional and post-translational control of AP2/ERFs were discovered. The expression level of most AP2/ERFs was usually low under normal conditions, but the developmental transition, hormone, and stress stimulus would induce or repress their expression at a specific stage, such as dehydration and heat-induced DREB2A in *Arabidopsis* [7,8]. The stability of DREB2A was also affected by phosphorylating its negative regulatory domain through casein kinase1 [9] while the N terminal methionine of RAP2.12 was removed through ubiquitin-mediated protein degradation [10]. Multifaceted control of AP2/ERFs implied their functional importance and possibly ensure an effective response. Many studies have reviewed the advances of AP2/ERFs in plants, summarizing their function, regulatory mechanisms, and evolution [11,12], specifically discussing their functions in *Arabidopsis* [5] and potential breeding practices in rice [6]. However, few studies have reviewed the characteristics of AP2/ERFs in the maize genome although few members of this family have been functionally characterized. In this review, we identified the AP2/ERFs in the newest reference genome, summarized the cloned genes associated with development and stress response (Table 1), and speculated their potential functions and interacting networks in maize. We also discussed the application of AP2/ERFs in the breeding process of maize.

## 2. The Characteristics of AP2/ERFs in Regulating Gene Expression in Plants

TFs regulate target gene expression through their conserved DNA binding domain [12,33,34,35,36,37], and the EREB domain of AP2/ERFs seemingly exhibits subfamily-specific binding activity of target cis-elements in the gene promoter region [3]. ERF subfamily members preferentially recognize to ethylene-response element (ERE) with the AGCCGCC core sequence to respond to ethylene, pathogens and wounding [38,39,40], and DREBs bind to Dehydration-Response or C-Repeat Element (DRE/CRT) with the A/GCCGAC core sequence that localizes within genes in response to ABA, drought, and cold [41,42]. However, the members of both families have been reported to bind to both ERE and DRE/CRT [43] while some novel DNA elements were also found in binding regions of AP2/ERFs such as OsDREB1C in rice and group VII members of AP2/ERFs (ERF-VIIs) in *Arabidopsis* [44,45,46,47,48,49,50], indicating the DNA binding diversity of AP2/ERFs. The effect of AP2/ERFs on target genes, activation or repression, is dependent on their conserved motif beside the EREB domain, and the repression domain of AP2/ERF proteins in *Arabidopsis* has been reviewed [51], in which the EAR motif with the consensus sequence LxLxL or DLNxxP was the main category [52,53]. These proteins containing EAR motifs can interact and recruit TOPLESS or histone modifiers to co-repress the expression of target genes [54,55,56]. Moreover, AP2/ERF proteins can interact with transcriptional regulators and structural proteins to form stable complexes, which determine their function, including localization, stability, abundance, transcriptional activity, and target specificity [51]. This is exemplified by the interaction of acyl-coA binding proteins (ACBPs) with ERF-VIIs via an ankyrin-domain in the plasma membrane, which prevented ERF-VIIs to enter the cytosol and nucleus [57,58].

In addition to directly regulating genes involved in the development, biotic, and abiotic stress, AP2/ERFs also mediated hormone signaling, including stress-associated hormones abscisic acid (ABA) and ethylene (ET) and growth-related hormones gibberellic acid (GAs), cytokinin, and brassinosteroid (BRs). ABA insensitive 4 (ABI4) which belongs to the DREB subfamily is a key component in the ABA signaling pathway, stabilized by stress, ABA, and phosphorylation [59]. ERF18 also activated the PP2C family phosphatase gene ABI2 to inhibit ABA signaling [60]. AP2/ERFs are downstream regulatory factors of ET signaling, which also control ET homeostasis through a negative feedback mechanism. ERF-VIIs play central roles in response to flooding stress, in which RAP2.2 was induced by hypoxia-promoted ET but the expression of ACSs that synthesize ET was decreased in RAP2.2 overexpression lines and increased in its mutant [61]. Furthermore, the SUB1A in rice promoted the accumulation of SLR1 and SLRL1 to repress GA signaling, which inhibited the internode elongation [62,63,64,65], and SK1/SK2 resulted in increasing GA 20-oxidases, which facilitated internode elongation [66,67]. Moreover, SUB1A activated BR biosynthesis and signaling to induce GA 2-oxidase 7 to degrade GAs, implying the cross-talk between GA and BR under submergence tolerance [68].

## 3. Comprehensive Identification of AP2/ERFs in Maize

The reference genome of maize assembled from inbred line B73 has been updated and reannotated with the development of sequence techniques, expectedly increasing the continuity and quality. The commonly used reference genome was B73 5b+, B73 version 4 (v4), and B73 v5. However, few studies had been conducted to integrate the member of AP2/ERF in these three versions, limiting further investigation of their functions. To identify the AP2/ERF members in maize, a total of 212 AP2/ERF genes named *ZmEREB1* to *ZmEREB212* were downloaded from Grassius (www.grassius.org, accessed on 23 November 2022), which were based on the genome of B73 5b^+^. These identities were further transformed into corresponding gene models within the genome of B73 v4 and v5. The keyword “EREB” was also applied to search AP2/ERF genes in MaizeGDB (www.maizegdb.org, accessed on 23 November 2022) and results demonstrated that another 28 ZmEREB genes (from *ZmEREB213* to *ZmEREB240*) were discovered. All of the proteins in B73 v4 and v5 were subjected to the Pfam database to identify the AP2/ERF domains, which were used to verify the structural domain of *ZmEREB1* to *ZmEREB240*. The gene named *ZmEREB234* in MaizeGDB was not detected in the AP2/ERF domain using the Pfam database, and *Zm00001d005203* which was only detected in B73 v4 was renamed as *ZmEREB234*. Finally, all 240 AP2/ERFs in maize were comprehensively checked and confirmed, with corresponding gene models in the B73 genome of 5b^+^, v4, and v5 (Table S1). Of 240 members, *ZmEREB35*, *ZmEREB43*, *ZmEREB143*, and *ZmEREB221* have two copies in B73 v5 while *ZmEREB120* has three copies. Three genes (*ZmEREB73*, *ZmEREB99*, and *ZmEREB194*) were only detected in B73 5b^+^, ZmEREB70 was not detected in B73 v5, six genes (*ZmEREB37*, *ZmEREB75*, *ZmEREB213*, *ZmEREB225*, *ZmEREB215*, and *ZmEREB231*) were not detected in B73 v4, and eleven genes (*ZmEREB217*, *ZmEREB221*, *ZmEREB224*, *ZmEREB226*, *ZmEREB227*, *ZmEREB228*, *ZmEREB230*, *ZmEREB233*, *ZmEREB236*, *ZmEREB237*, and *ZmEREB239*) were not detected in B73 5b^+^. These results suggested that the updated genome of B73 v4 and v5 have more annotated members of AP2/ERFs, and the comprehensive list identified here would promote functional investigation.

## 4. AP2/ERFs Regulating the Development Process in Maize

Maize is an important cereal crop that mainly yields grains to maintain food safety and energy supply, and inflorescence architecture and development determine its final production. *Branched silkless1* (*bd1*), belonging to AP2/ERFs family member, was firstly cloned in maize, specifically expressing in ear and tassel tissue [16]. Mutants of *bd1* appeared as indeterminate spikelets and yielded numerous lateral spikelets, which were positively associated with the expression level of *bd1*. In mutants, spikelet meristems failed to initiate an outer glume and produced spikelet pair meristems. BD1 repressed indeterminate branching in both inflorescences whereas the loss of BD1 has different effects in the ear and tassel, conserving in different grasses. Teosinte is an ancestor of maize, providing valuable alleles in the maize breeding process. The recombinant inbred lines derived from the crossing of B73 and *Zea diploperennis* were applied to identify candidates associated with differential traits between maize and teosinte [13]. Two AP2/ERF family members, *ZmEREB92* and *ZmEREB93*, were screened as key candidate genes regulating ear height and the ratio of the ear to plant height. Further studies on how these two genes regulate plant height will be conducted. Crown roots of maize play necessary roles in water and nutrient acquisition and lodging tolerance. *ZmRAP2.7* is expressed in all types of roots, and the protein is localized in the nucleus, the mutant of which displayed lessened expression and a reduced number of brace roots [15]. The mutant line, *Corngrass1*, with increasing expression of *ZmRAP2.7*, had an increased number of brace roots. An SNP variation at the fifth exon of *ZmRAP2.7* in the maize association panel showed an association with the number of brace roots. *ZmEREB94* is highly expressed in the maize endosperm and ZmEREB94-GFP fusion protein is localized in the nucleus, showing transcriptional activation activity [14]. ZmEREB94 could regulate *ZmSSI*, *ZmSh2*, and *ZmGBSSI* expression to affect starch synthesis in maize. These investigations provide clues about AP2/ERFs in regulating root, inflorescence, and grain development, and exploring their alleles will uncover their genetic mechanism and create new germplasms to achieve breeding goals.

## 5. AP2/ERFs Involved in Abiotic Stress in Maize

Drought stress is one of the most serious stresses impacting maize growth, and at least five AP2/ERFs in maize (Table 1) have been shown to be involved in drought response. *ZmEREBP60* is a positive regulator under drought stress. Expression of *ZmEREBP60* was strongly induced by drought in the roots, coleoptiles, and leaves of maize, which localized subcellularly into the nucleus [21]. Overexpressed lines of *ZmEREBP60* showed increased tolerance to drought stress through reducing H_2_O_2_ accumulation and malondialdehyde content. Transcriptome analysis of transgenic lines of *ZmEREBP60* demonstrated that the expression of genes involved in H_2_O_2_ catabolism, water deprivation response, and the abscisic acid signaling pathway were differentially regulated. The cuticular wax on the surfaces of plants is an important component for resisting adverse environmental conditions. Ectopic expression of *ZmEREB46* promoted the accumulation of epicuticular wax on the leaves and increased the drought tolerance in *Arabidopsis* while the amount of epicuticular wax of maize leaves in the *ZmEREB46* knockout mutant decreased by approximately 50% compared to the wild-type [20]. ZmEREB46 could directly bind to promoter regions of *ZmCER2*, *ZmCER3.2*, and *ZmKCS1* to affect the biosynthesis of very long-chain waxes and be involved in the cutin biosynthesis pathway. *ZmERF21* was induced by polyethylene glycol treatment and is highly expressed in the root and leaf [27]. The mutant line of *ZmERF21* showed a sensitive phenotype of drought stress in maize whereas the overexpression line enhanced tolerance through physiological changes. *ZmERF21* can directly bind three cis-elements of GCC(A/T/C/G)CCG, TGGCCAC, and GAAAATAGC(A/T/C/G)ATG, which are located within the promoter region of genes related to hormone (ET and ABA) and calcium signaling as well as other stress-responses. Drought stress slightly upregulated the expression of *ZmDREB2.7* in the leaves and roots of maize, and ZmDREB2.7 protein showed a high level of transactivation activity [22]. Natural variations of *ZmDREB2.7* were significantly associated with drought tolerance, and gene expression of *ZmDREB2.7* was positively correlated with maize survivability under moderate/early drought stress. ZmDREB2.7 can specifically bind both typical DRE sequences, but had a low level of binding affinity of the GCC sequence. Overexpression lines of *ZmDREB2.7* in *Arabidopsis* and maize enhanced drought tolerance at the seedling stage. Overexpressed lines of *ZmDBF1* in *Arabidopsis* showed different degrees of growth retardation, which correlated with the expression level of *ZmDBF1* [26]. A higher percentage of seed germination in transgenic *Arabidopsis* in comparison with control plants was observed. ZmDBF1 specifically binds DRE cis-elements and interacts with ZmDIP1; this interaction was essential for the nuclear localization of DIP1. Co-transfection of DBF1 with DIP1 enhanced promoter activity of rab17 in the absence of ABA treatment.

Besides responding to drought stress, maize AP2/ERFs are also involved in salt, waterlogging, and cold stress. *ZmEREB20* is a positively responsive gene under salinity conditions [19]. The overexpression of *ZmEREB20* in *Arabidopsis* demonstrated increased ABA sensitivity and delayed seed germination after salt stress. *ZmEREB20* regulated the expression of genes related to ABA and GA, and transgenic lines presented higher survival rates and elevated ROS scavenging. Moreover, overexpression lines enhanced the expression of auxin-related genes, ion transporter genes, and root hair growth to regulate root hair plasticity. ERF-VIIs play important roles in plant responses to flooding. A candidate gene association analysis showed that *ZmEREB180* belonging to maize ERF-VIIs was significantly associated with waterlogging tolerance, and its expression responded to waterlogging and was up-regulated by ethylene [25]. The variations in the 5′-untranslated region (5′-UTR) were significantly associated with phenotypes after waterlogging stress, and the expression level of *ZmEREB180* under waterlogging conditions also correlated with phenotypes. Overexpressing of this gene in *Arabidopsis* and maize could enhance tolerance to waterlogging stress. Overexpressed lines in maize had higher amounts of adventitious roots (ARs) and higher antioxidant levels, which were consistent with the expression of genes involved in AR development and reactive oxygen species homeostasis. The expression of *ZmCBF3* (*ZmEREB3*) was root-specific, and the 234-bp fragment upstream of the *ZmCBF3* gene exhibited the highest level of GUS activity in transgenic *Arabidopsis* [17]. The cold stress-activated *ZmCBF3* promoter and element of CANNTG were responsible to respond to cold stress.

Part of AP2/ERFs in maize are involved in diverse stress conditions (Table 1). The ZmDREB1A proteins are specifically bound to the DRE cis-acting element (G/ACCGAC), and their expression of it was induced by cold and high-salinity stress [18]. Overexpression lines of *ZmDREB1A* in *Arabidopsis* increased the expression of target stress-inducible genes, and the transgenic plants exhibited higher tolerance to drought and freezing. The promoter sequence of *ZmERF1* (*ZmEREB148*) contains many cis-regulatory elements related to stress responses, and ABA and ethylene treatment in maize increased its expression [23]. Transgenic lines of *ZmERF1* in *Arabidopsis* displayed enhanced salt tolerance, and drought and heat resistance compared with the WT plants while tolerance-related genes were also up-regulated. Expression levels of *ZmEREB160* are significantly induced by the treatment of PEG6000, NaCl, and ABA, and overexpressed *ZmEREB160* in *Arabidopsis* increased tolerance to osmotic and ABA stress [24]. Seedlings of transgenic lines had longer roots under ABA and mannitol treatments and had elevated survival rates under drought stress. Expression of ABA/drought stress-related genes, including *ABI2*, *ABI5*, *COR15*, *DREB2A*, and *RD29B*, were up-regulated in overexpressed lines.

## 6. AP2/ERFs Mediating Biotic Stress in Maize

Maize terpenoid phytoalexins (MTPs) were induced by multiple pathogens and display extensive antimicrobial activities to suppress fungal growth [69,70]. The accumulation of MTPs was precisely regulated through MTP biosynthetic genes (MTGs), and a mutant defective in MTPs showed decreased resistance to *F. graminearum* [71,72]. Expression of *ZmEREB92* was induced by *F. graminearum* and also activated the expression of MTGs [32], indicating that *ZmEREB92* was a positive regulator in *MBG* expression. Mutants of *ZmEREB92* in maize showed significantly reduced resistance to *F. graminearum,* in which the induction of *MBGs* was delayed, thus decreasing MTP accumulation. ZmMYC2 interacted with and activated ZmEREB92 and the interaction complex directly binds to *MBG* promoters whereas ZmJAZ14 physically interacted with both ZmEREB92 and ZmMYC2 to inhibit their action. Collectively, *ZmEREB92* is involved in the regulation of JA/ET-mediated MTP accumulation upon *F. graminearum* infection. The mutant line of *ZmERF147* developed lesion areas after *F. graminearum* infection compared with B73, and the expression levels of almost ZmPRs in the mutant were relatively low after 1 d inoculation, implying affecting *ZmERF147* resistance to *F. graminearum* in maize [29]. ZmMYC7, a ZmMYC2 ortholog, could directly bind to the G-box element (5′-CACGTG-3′) in the promoter of *ZmERF147* and interact with ZmJAZ8, ZmJAZ11, and ZmJAZ12, of which *ZmMYC7* mutants downregulated the expression levels of the defense-associated genes such as *ZmPR1*, *ZmPR2*, *ZmPR3*, *ZmPR5*, *ZmPR6*, and *ZmPR7* in response to *F. graminearum* infection. Another two AP2/ERF members, *ZmERF105* and *ZmERF061*, were detected to respond *Exserohilum* (*E.*) *turcicum. ZmERF105* contains an AP2/ERF domain and a conserved LSPLSPHP motif in its C-terminal region [28]. Expression of *ZmERF105* responded to the treatment of *E. turcicum*, its protein localized to the nucleus, and it directly binds to GCC-box elements. Overexpression of *ZmERF105* in maize increased resistance to *E. turcicum*, and the activities of superoxide dismutase and peroxidase in transgenic lines were higher than in wild-type lines. ZmERF105 could enhance the expression of pathogenesis-related (PR) genes such as *ZmPR1a*, *ZmPR2*, *ZmPR5*, *ZmPR10.1*, and *ZmPR10.2* after infection with *E. turcicum*. Mutant lines of *ZmERF105* showed opposite phenotypes and gene expression with overexpression lines. *E. turcicum* inoculation significantly induced the expression of nucleus-localized *ZmERF061* (*ZmEREB61*) and it was also induced by salicylic acid (SA) and methyl jasmonate treatments [31]. zmerf061 mutant lines were more sensitive to *E. turcicum.* In mutant lines, expression of defense-related genes *ZmPR10.1* and *ZmPR10.2* and JA signaling-related gene *ZmLox1* decreased while the expression of the SA signaling-related gene *ZmPR1a* increased after *E. turcicum* infection. ZmERF061 could interact with ZmMPK6-1.

Maize is a model system for the investigation of indirect plant defense against herbivores, and biosynthesis and emitting of sesquiterpenes through terpene synthases (TPS) is one way to attract the natural enemies against herbivores. The promoter of *TPS10* retained necessary and sufficient elements for its herbivore responsiveness, and (Zm)*EREB58* responded to herbivory and jasmonate, having similar expression patterns with *TPS10* [30]. EREB58 directly binds to GCC-box within the *TPS10* promoter region to promote its expression through in vivo and in vitro experiments. Overexpressing *EREB58* in maize enhanced the accumulation of the *TPS10* transcript and two major TPS10-catalyzed sesquiterpenes whereas transgenic lines of EREB58-RNAi abolished the induction expression of *TPS10* and its volatiles.

## 7. Maize AP2/ERFs Functioning in Diverse Biological Processes

Compared with *Arabidopsis* and rice, the investigation of AP2/ERFs in maize has still lagged seriously. However, the expression proof from transcriptomic data has also demonstrated that lots of AP2/ERFs in maize are involved in multiple biological processes, including development and stress response. Transcriptome analysis of cold- and heat-treated seedlings of B73 lines showed that a lot of AP2/ERFs responded to temperature stress, in which many genes were commonly up-regulated in both conditions, specifically for DREB subfamily members [73]. The time-course expression response of maize seeding roots under waterlogging conditions showed that many AP2/ERFs were induced, in which eight genes belong to ZmERF-VII [74]. Several members of AP2/ERFs were also detected by heavy metal Pb pollution [75]. Five AP2/ERFs in response to low nitrogen stress in wild-type plants were identified [76], implying their potential function in nitrogen metabolism. Four members responded to *Fusarium verticillioides* infection, which causes ear rot in maize [77]. AP2/ERFs were also differentially expressed in maize inbred lines with a differential abundance of inositol phosphates [78]. Moreover, numerous studies based on comparative transcriptomes have been conducted to investigate regulatory networks responding to clod, salt, and aluminum, and large amounts of responsive AP2/ERFs were identified [79,80,81]. Taken together, maize AP2/ERFs are also involved in and mediated extensive biological activities, but their definite roles of them need to clarify in the future.

## 8. The Potential Roles of Maize AP2/ERFs Inferring from Rice Homologs

**Figure 1:**
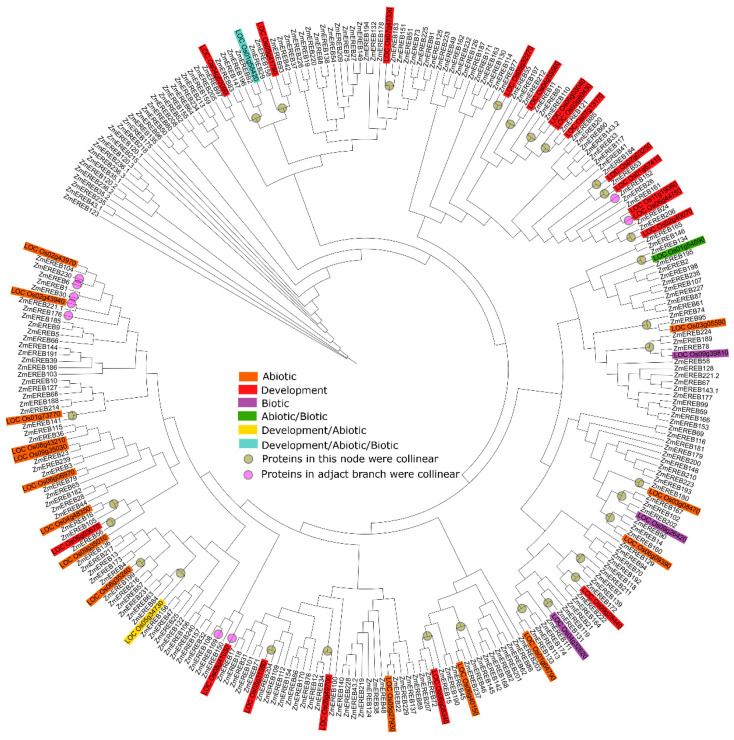
The phylogenetic tree of AP2/ERFs in maize and rice using MEGA11 software based on the neighbor-joining method. All members in maize and rice-cloned AP2/ERFs were subjected to construct the tree, and the bootstrap value was set as 1000. The rectangular box with different colors represented the development and stress processes that involved by rice AP2/ERFs, and the circle with different colors represented the collinear relationship of AP2/ERFs between rice and maize.

The homolog proteins within a species or among different species have evolved the conserved domains and motifs, which determine their similar functions. Maize and rice belong to monocotyledonous plants of the gramineous family and have a close relationship. The homologs in maize and rice such as the convergently selected gene of *KRN2* [82] always play similar roles in regulating biological activity. To dissect the potential roles of AP2/ERF genes in maize, rice-cloned AP2/ERFs (reviewed by [4]) and all maize AP2/ERFs were subjected to construct a phylogenetic tree using MEGA11 [83]. Furthermore, the gene synteny of AP2/ERFs between maize and rice were also calculated using MCScanX [84]. A total of 40 AP2/ERFs in rice were applied to align homologs in maize, and the closest maize genes were discovered (Figure 1). *OsERF71* (*LOC_Os06g09390*) was a drought response gene in rice [85], and its homolog *ZmEREB160* also regulated drought tolerance in maize [24]. *FZP* (*LOC_Os07g47330*) prevented the formation of axillary meristems in rice spikelets [86] while its homolog *ZmEREB151* determined the identity of the spikelet meristem [16]. These data confirmed the close roles of homologs in maize and rice. Interestingly, most of these homologs (*OsERF71* and *ZmEREB160*; *FZP* and *ZmEREB151*) were collinear (Figure 1), implying that AP2/ERFs in maize and rice may be a co-evolution. These results will facilitate the functional assignment of AP2/ERFs in maize.

## 9. The Putative Regulatory Network of Maize AP2/ERFs

**Figure 2:**
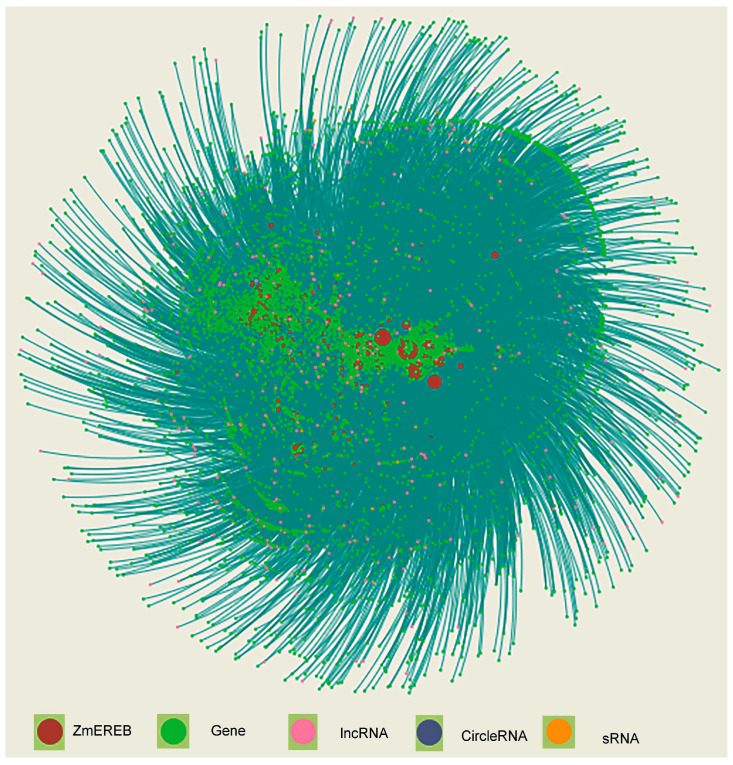
The integrative networks mediated by AP2/ERFs.

The transcriptional regulatory networks in the eukaryotic cell were mediated by diverse TFs and cis-regulatory elements (CREs). With the development of molecular techniques and bioinformatic pipelines, large-scale TFs and CREs were identified in plants, including the CREs code of stress response in *Arabidopsis* [87] and predicting expressional response to cold stress across species [88]. Maize is an ideal genetic system to dissect the TFs and CREs, and lots of studies have been recently conducted to investigate the transcriptional maps based on experimental, computational, and integrative approaches in maize with tissue and single-cell resolution [89,90,91,92,93,94,95]. AP2/ERFs have plentiful members in the maize genome, regulating diverse developmental processes and stress response, most of which were found to regulate gene expression through the binding of a target promoter sequence (Table 1). Using ATAC-seq and ChIP-seq experiments, the binding sites of 14 AP2/ERFs were globally identified, in which the conserved motif AGCCGCC was discovered [89]. Combined with a predicting model, many novel CREs were putatively bound by these genes, implying wide-range regulation through AP2/ERFs [89]. The largest integrated genomic networks in maize were constructed through the collection of chromatin interaction data, (translatomic) coexpression data across different tissues and stages, and interactome, which included 32,758 protein-coding genes, 4013 lncRNAs, and 183 circle RNAs [95]. To construct the highly confident networks mediated by maize AP2/ERFs, the ‘Network Creation’ module (http://minteractome.ncpgr.cn/, accessed on 27 February 2023) was applied to retrieve the co-expressed elements. A total of 55,706 interactions through 201 AP2/ERFs, 15,702 protein-coding genes, 60 sRNAs, 1033 lncRNAs, and 32 circle RNAs were detected (Figure 2). These data demonstrated that most maize AP2/ERFs are co-expressed with approximately 50% of protein-coding genes in integrative networks. Furthermore, these AP2/ERFs also co-expressed with the large amount of non-coding RNAs such as sRNA, lncRNA, and circleRNA, indicating their essential roles in the maize genome.

**Figure 3:**
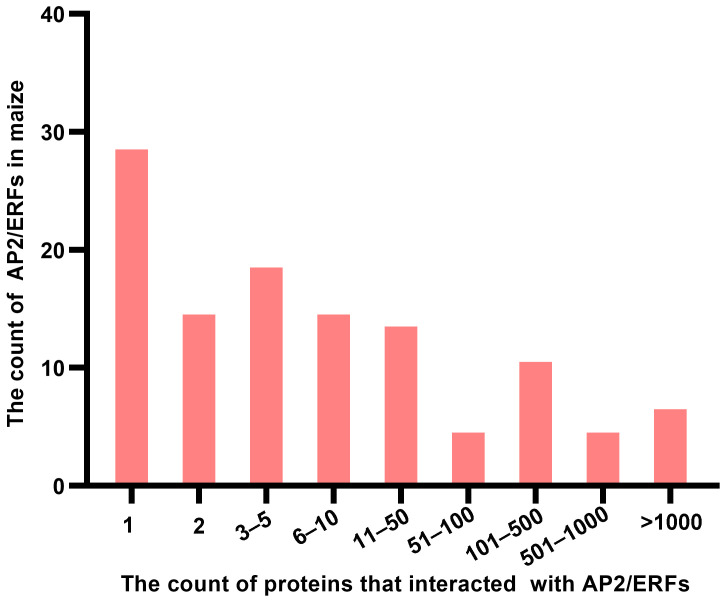
The number of AP2/ERFs and their partners in maize.

Moreover, the benefit of the large-scale screening yeast libraries, the potential partner of maize AP2/ERFs could be obtained [95]. Using maize AP2/ERF genes as a query, a total of 21,228 interactions were discovered, of which 120 AP2/ERFs interacted with 9820 proteins. Most of the AP2/ERFs can interact with more than one protein, and some of them can interact with more than 100 proteins (Figure 3), indicating that AP2/ERFs mediated the complex networks, although experimental verification will further be needed. Moreover, part of AP2/ERFs can interact with itself or other AP2/ERFs such as ZmEREB94 interacting with itself and ZmEREB160, implying that AP2/ERFs needed to form homodimers or heterodimers to function well. These predicted partners are the vital data source for dissecting the function of maize AP2/ERFs.

## 10. Perspectives

**Figure 4:**
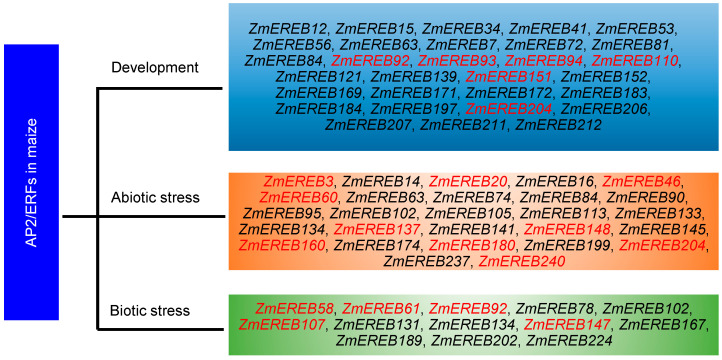
The candidate AP2/ERFs are putatively involved in the development, abiotic and biotic stress. The genes with red color represented the cloned category in maize, and the genes with black color represented the predicted category based on rice homologs.

Maize is an upland cereal crop, suffering diverse stresses in its life cycle. Stable and high yields of maize are always the targeted goals of the breeding process. Map-based cloning of genes associated with maize ear length has demonstrated that a gene *ZmACO2* controlling ethylene synthesis was identified to determine ear length, flower number, and fertility, regulating grain yield per ear in hybrids lines [96]. Recent de novo assembly of 12 founder inbred lines also showed that structural variations of *ZmACO2* contribute to yield heterosis in maize [97]. These investigations indicated that ethylene plays a vital role in the yield production of maize. However, little ethylene-mediated regulation of maize yield and heterosis is known. As the downstream signaling of ethylene, AP2/ERFs are key candidates for these processes. Clarifying the roles of AP2/ERFs involved in yield and heterosis will provide potential targets for genetic improvement. Ethylene is also a stress response hormone, specifically responding to flooding/hypoxia. The investigation of hypoxia in *Arabidopsis* has manifested that ERF-VIIs, a subgroup member of AP2/ERFs, are core elements sensing hypoxia in plants [10]. The homologs in rice and *Arabidopsis* have shown enhanced tolerance to flooding stress, such as *HRE1/HRE2*, *SUB1A*, and *SK1/SK2* [66,98,99]. A total of 19 ERF-VIIs were discovered in the maize genome and only one of them was verified to regulate waterlogging tolerance [25]. There are large gaps that will need to be filled since maize is also sensitive to flooding. Moreover, two interacting AP2/ERFs (ERF95 and ERF97) of *Arabidopsis* regulate basal thermotolerance [100], providing direct evidence of heat stress. However, few AP2/ERFs in maize were found to regulate thermotolerance. Based on the function and collinearity of AP2/ERFs in rice, a total of 49 homologs in maize were discovered, in which 24, 17, and 8 AP2/ERFs were putatively involved in development, abiotic stress, and biotic stress (Figure 4). These categories would accelerate the functional analysis of AP2/ERFs in maize and facilitate their applications in the genetic breeding process.

## Figures and Tables

**Table 1:** The information of cloned-AP2/ERFs in maize.

Gene Name	Bias Name	Category	Traits	Reference
*ZmEREB92*		Development	Ear height and the ratio of ear-to-plant height	[13]
*ZmEREB93*		Development	Ear height and the ratio of ear-to-plant height	[13]
*ZmEREB94*		Development	Starch synthesis	[14]
*ZmEREB110*	*ZmRAP2.7*	Development	Brace roots development	[15]
*ZmEREB151*	*BD1*	Development	Identity of the spikelet meristem	[16]
*ZmEREB3*	*ZmDREB1A, ZmCBF3*	Abiotic stress	Cold, salt, drought	[17,18]
*ZmEREB20*		Abiotic stress	Salt	[19]
*ZmEREB46*		Abiotic stress	Drought, biosynthesis of leaf epicuticular	[20]
*ZmEREB60*	*ZmEREBP60*	Abiotic stress	Drought	[21]
*ZmEREB137*	*ZmDREB2.7*	Abiotic stress	Drought	[22]
*ZmEREB148*	*ZmERF1*	Abiotic stress	Salt, drought, and heat	[23]
*ZmEREB160*		Abiotic stress	Drought	[24]
*ZmEREB180*		Abiotic stress	Waterlogging	[25]
*ZmEREB204*	*ZmDBF1*	Abiotic stress	Osmotic	[26]
*ZmEREB240*	*ZmERF21*	Abiotic stress	Drought	[27]
*ZmEREB107*	*ZmERF105*	Biotic stress	*E. turcicum*	[28]
*ZmEREB147*	*ZmERF147*	Biotic stress	*F. graminearum*	[29]
*ZmEREB58*		Biotic stress	Herbivory	[30]
*ZmEREB61*	*ZmERF061*	Biotic stress	*E. turcicum*	[31]
*ZmEREB92*		Biotic stress	*F. graminearum*	[32]

## Data Availability

Not applicable.

## Supplementary Materials

The following supporting information can be downloaded at: https://www.mdpi.com/article/10.3390/ijms24065416/s1.
